# Optimization of the Microwave-Assisted Extraction of Caffeine from Roasted Coffee Beans

**DOI:** 10.3390/foods13152333

**Published:** 2024-07-24

**Authors:** Ivan M. Savić, Ivana M. Savić Gajić, Dragoljub G. Gajić

**Affiliations:** 1 Faculty of Technology in Leskovac, University of Nis, Bulevar Oslobodjenja 124, 16000 Leskovac, Serbia; savicivana@tf.ni.ac.rs; 2 School of Electrical Engineering, University of Belgrade, Bulevar Kralja Aleksandra 73, 11000 Belgrade, Serbia; office@jakako.tech; 3 Jakako Doo, Hadži-Đerina 16, 11000 Belgrade, Serbia

**Keywords:** caffeine, coffee beans, microwave-assisted extraction, optimization, Box–Behnken design, UHPLC-ESI-MS/MS analysis

## Abstract

This study aimed to develop a fast procedure for caffeine extraction from roasted coffee beans. The microwave-assisted extraction was carried out in the microwave oven with an operating frequency of 2450 MHz. The response surface methodology based on a Box–Behnken design was used to model and optimize the extraction process. Among the analyzed extraction parameters (factors), the influence of extraction time (2–6 min), liquid-to-solid ratio (5–15 mL/g), and microwave power (336–595 W) were considered, while the yield of extracted caffeine was observed as the response of the system. Water was used as the solvent of choice for the extraction of caffeine. The optimum conditions were as follows: extraction time, 2 min; liquid-to-solid ratio, 15 mL/g; and microwave power, 500 W. In this optimized condition, the expected extraction yield of caffeine was 1.01 g/100 g dry weight (value confirmed by experimental assays). The total energy consumed of 1.7 kWh/100 g of purified caffeine indicated a more energy-efficient procedure by about 1200–15,000 times than the reported procedures. This study showed that caffeine can be quantitatively extracted from roasted coffee beans through a green approach and that the isolated caffeine has a high purity degree, which was confirmed by the UHPLC-ESI-MS/MS method. With this quality, isolated caffeine could be further used as an active ingredient in the food industry, while for pharmaceutical purposes, it must be further purified.

## 1. Introduction

Coffee is one of the world’s most popular and most consumed beverages after water. The prediction of the global annual production of coffee in 2023/24 is to reach 171.4 million bags higher than the previous year [1]. Coffee beans consist of over 1000 bioactive substances such as phenolic compounds, polysaccharides, lipids, proteins, fatty acids, caffeine, trigonelline, and free amino acids [2]. The composition of coffee beans can vary depending on their type, cultivation, harvesting, storage, and processing. Traditionally, the beneficial effects of coffee originate exclusively from caffeine, but the other compounds also contribute to its valuable properties [3].

Caffeine, as the main alkaloid, is present in coffee beans in an amount of 1–4% of its dry weight and its content can vary significantly between varieties [4]. Due to its toxicity against insects and other pests, the plants use this compound as a natural pesticide for their protection [5]. It is a natural stimulant with expressed antioxidant properties that has numerous beneficial effects on human health. It slows down the aging of the skin by neutralizing the negative effects of UV radiation and by accelerating the overall metabolism (especially microcirculation). It prevents the deposition of fat, even the formation of cellulite, and promotes alertness [6]. The negative effect of coffee on human health is mainly related to the caffeine concentration. The largest part (80%) of the produced caffeine is used in the food industry, while somewhat less in the pharmaceutical and cosmetic industries. On the market, most food products that may contain caffeine are energy drinks, chocolate, soft drinks, ice creams, puddings, etc. [7].

Caffeine isolation from coffee beans is a multistep process consisting of solid–liquid and liquid–liquid extractions due to the complex sample. The solid–liquid extraction of caffeine with different solvents such as water, alcoholic solutions, chloroform, dichloromethane, acetone, hexane, ethyl acetate, carbon tetrachloride, or their combination is used to avoid the extraction of cellulose from this plant material [8]. “Green solvents” including eutectic solvents [9] and ionic liquids [10] are also used for the extraction of caffeine. Considering practical, ecological, and economic reasons, water is a more suitable solvent than other solvents. The better solubility of caffeine in water compared to alcoholic solutions can be justified by its hydrophilic nature. It can form hydrogen bonds with water molecules, while the number of hydrogen bonds formed, for example, with ethanol solution would be smaller [11].

Among the solid–liquid extractions, maceration [10] and Soxhlet extraction [12] are the most commonly used conventional extraction techniques. However, following the concept of green chemistry, which is based on the application of clean technologies, intensive work was done to determine the possibility of applying modern extraction techniques, such as ultrasound-assisted extraction [8], microwave-assisted extraction (MAE) [13], supercritical carbon dioxide extraction [14], or a combination of these methods for caffeine isolation from coffee. After solid–liquid extraction, the obtained extract contains caffeine and other bioactive compounds such as polyphenols, amino acids, saponins, pigments, etc. [15]. Liquid–liquid extraction with chloroform is used for caffeine isolation from the complex mixture due to its good solubility [16]. This solvent creates an emulsion layer that is difficult to separate, so methylene chloride and dichloromethane are used [10]. The low solubility of caffeine in ethanol was used for its precipitation, i.e., purification [17].

MAE has proven to be one of the most promising extraction techniques among the available techniques for caffeine extraction from coffee beans and spent coffee grounds [18]. It is considered an advanced and sustainable alternative to conventional and other green extraction techniques due to many advantages: faster energy transfer, reduction of extraction time and solvent use, higher selectivity, and increased yield [19]. The MAE of caffeine from coffee or its grounds was carried out using different types of microwave equipment and operating conditions. Frequently analyzed extraction parameters (factors) are microwave power, extraction temperature, extraction time, and type of solvent to achieve the highest caffeine yield [18]. In the literature, response surface modeling (RSM) is a useful mathematical tool for determining the optimal factor levels and the interactions between the analyzed factors of MAE [20]. According to our knowledge, RSM has been insufficiently used to optimize the MAE of caffeine from coffee or its grounds. Lopes et al. [13] optimized the closed-vessel MAE system for caffeine extraction from roasted coffee using a Box–Behnken design (BBD).

The continual development of advanced and innovative procedures for caffeine isolation from coffee beans is still an attractive research topic in the scientific community. The isolation process is not carried out to reduce the caffeine content due to its toxic effect in coffee, but to obtain a pure active substance that can be used to prepare various food and pharmaceutical products. Respecting the green technology concept which includes using energy-efficient procedures and non-toxic, eco-friendly, biodegradable, and cheap solvents, this study aimed to apply the open-vessel MAE system with water as a solvent of choice in the solid–liquid extraction step. To define the optimal levels of analyzed factors, the modeling and optimization of the procedure were carried out using the RSM based on the BBD according to the maximum caffeine yield. Thanks to this approach, savings in the consumption of available resources were achieved due to the smaller number of performed extractions required to optimize the analyzed process. Also, the interactions between the extraction parameters were defined, which is impossible to do using a one-variable-at-a-time approach. The structural characterization of isolated caffeine was analyzed by the Fourier-transform infrared (FTIR) method. At the same time, its purity was estimated using the ultrahigh-performance liquid chromatography–tandem mass spectrometry with electrospray ionization (UHPLC-ESI-MS/MS) method.

## 2. Materials and Methods

### 2.1. Chemicals and Reagents

Caffeine (purity of 99.7%) (Alfa Aesar, Karlsruhe, Germany), 96% (*v*/*v*) ethanol (Zorka, Sabac, Serbia), dichloromethane (Sigma-Aldrich Chemie GmbH, Taufkirchen, Germany), acetonitrile and water (HPLC-MS grade) (Sigma Chemical, St. Louis, MO, USA) were used in this study.

### 2.2. Caffeine Extraction from Roasted Coffee Beans

Roasted coffee beans were purchased from a local grocery store and then subjected to mechanical pretreatment (grinding) in an electrical mill (Braun Aromatic KSM2, Kronberg im Taunus, Germany) and sieved through a vibrating test sieve shaker. The coffee fraction with a particle size of 0.5 mm was further used for microwave-assisted extraction of caffeine. This caffeine isolation procedure consisted of two phases: solid–liquid and liquid–liquid extractions. In the first stage, a coffee sample (150 g) was treated with distilled water (900 mL) to which sodium carbonate (60 g) had previously been added. Water was the solvent of choice for caffeine extraction because its solubility in boiling water is higher compared to its solubility in ethanol [21].

The salt addition was necessary to convert poorly soluble compounds into water-soluble salts. The solid–liquid extraction was performed in a modified kitchen microwave oven (Vivax MWO-2070 BL) with a frequency of 2450 MHz. The experimental conditions for different extractions were defined according to the matrix of the used BBD. The RSM enabled the simultaneous monitoring of the effect of all analyzed factors. Among the extraction parameters (factors), the extraction time (2–6 min), liquid-to-solid ratio (5–15 mL/g), and microwave power (336–595 W) were analyzed. The extraction temperature of the system was 100 °C and maintained constantly because the water boiled in all experimental series under given extraction conditions. Caffeine yield was defined as the system response that was further maximized during the optimization process. Each extraction parameter was analyzed at three factor levels whose actual and coded values are given in Table 1. The factor levels were coded according to Equation (1):

(1) $$x=\frac{\left(X_{i}-X_{0}\right)}{∆X}$$

where *x* is the coded value, *X_i_* is the corresponding actual value, *X*_0_ is the actual value in the central domain, and Δ*X* is the increment *X_i_*, corresponding to a variation of one unit of *x.*

**Figure 1:**
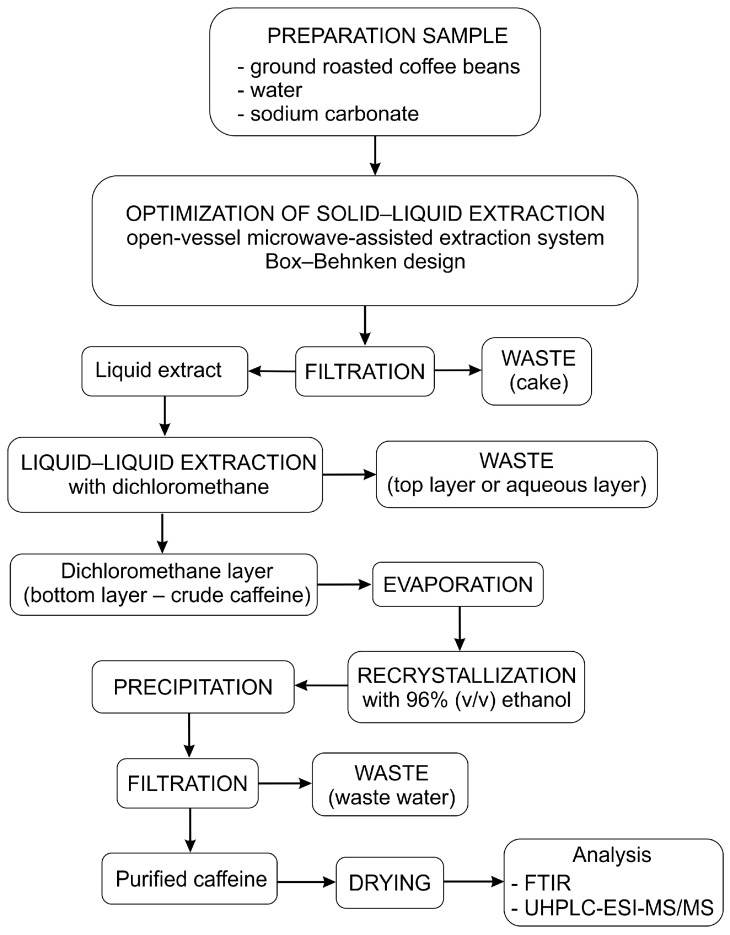
Process chain of caffeine isolation from ground roasted coffee beans.

After extraction, the extract was cooled under a stream of cold water, then filtered under a vacuum, and then centrifuged at 6000 rpm on a TH16B centrifuge (Hong Kong, China) for 15 min. In the second stage, the prepared extract was transferred to a separatory funnel and subjected to liquid–liquid extraction with dichloromethane to isolate caffeine. The extract was treated three times with 15, 15, and 10 mL of dichloromethane and slightly shaken to redistribute the caffeine between the two phases and establish an equilibrium state. The lower dichloromethane layer was washed twice with 20 mL of saturated sodium chloride solution to achieve better phase separation and then dried with anhydrous Na_2_SO_4_. Dichloromethane was separated from white crystals of raw caffeine (solid phase) using a vacuum rotary evaporator at 50 °C. Recrystallization of crude caffeine was carried out in 96% (*v*/*v*) ethanol solution. This stage involved heating the sample at the boiling temperature of the solvent and cooling it to +4 °C (the temperature achieved by storing it in a refrigerator). The process chain of extraction, isolation, and then purification of caffeine is presented in Figure 1.

To estimate the energy efficiency of the proposed procedure for caffeine isolation and compare it with other available literature, the total energy consumed (TEC) for the production of 100 g purified caffeine obtained under its optimal extraction conditions was calculated according to Equation (2):

(2) $$TEC=\frac{Microwave\;power\left(kW\right)\times Extraction\;time\left(h\right)}{Caffeine\;amount\;(100\;g)}$$

### 2.3. Caffeine Sample FTIR Analysis

The isolated caffeine was analyzed using FTIR spectroscopy. After preparation of KBr pellets, the FTIR spectra of isolated and standard caffeine were recorded in the wavenumber range from 4000 to 400 cm^−1^ with a resolution of 2 cm^−1^ on a spectrophotometer, Bomm Hartmann and Braun MB-series (Quebec, QC, Canada). The spectra were processed using Win-Bomem Easy software (http://www.ftirsearch.com/Features/Converters/Bomem_Win_Easy.asp?popup=no) (accessed on 20 July 2024) (Bomem GRAMS/32, Galactic Industries, Salem, NH, USA).

### 2.4. UHPLC-ESI-MS/MS Analysis

The UHPLC-ESI-MS/MS (Dionex Ultimate 3000 UHPLC+) method was used to evaluate the quality of the isolated caffeine. A Hypersil gold C_18_ column (50 mm × 2.1 mm, 1.9 µm) was thermostated at 40 °C. For chromatographic analysis, the isocratic elution program was used. The mobile phase consists of phase A (0.1% formic acid in water) and phase B (0.1% formic acid in acetonitrile) in a ratio of 85:15 (*v*/*v*). The flow rate of the mobile phase was adjusted at 0.2 mL/min. The diode array detector was recorded at wavelengths of 274 nm. The injected sample volume was 5 µL in the system. The sample was filtered through a 0.45 µm membrane filter (Merck Millipore, Burlington, MA, USA) before its injection into the system. The total analysis time was 5 min. The mass spectra were recorded in the positive ion single ion monitoring (SIM) mode at *m*/*z* 195. The mass detector was adjusted according to the following ion source conditions: source voltage, 4.5 kV; capillary voltage, 29 V; tube lens voltage, 75 V; capillary temperature, 275 °C; and sheath and auxiliary gas flow (nitrogen), 50 and 8 (arbitrary units). The used energy for collision-induced dissociation was 35 eV.

For the quantitative study, the caffeine standard was accurately weighed and dissolved in the mobile phase to obtain a reference stock solution (1 mg/mL). A series standard solution of caffeine in the concentration range of 1 to 100 μg/mL was prepared by dilution of the stock solution to construct the calibration curve. The calibration curve was constructed by plotting peak area (y) in the chromatogram obtained by recording using a mass detector against caffeine concentration (x, μg/mL). The limit of detection (LOD) and limit of quantification (LOQ) were determined based on signal-to-noise (S/N) ratios of 3 and 10, respectively. The sample of caffeine isolated from coffee beans was prepared at a concentration of 20 µg/mL to determine its purity compared to the available standard.

## 3. Results and Discussion

### 3.1. Modeling of Microwave-Assisted Extraction of Caffeine from Roasted Coffee Beans

The methodology implemented in this study is focused on optimizing the MAE of caffeine from roasted coffee beans, using water as a non-toxic solvent. The open-vessel MAE system was selected as the technique of choice due to the simplicity of the equipment, low operating costs, high extraction efficiency, and environmental protection requirements compared to other conventional techniques.

In order to minimize the consumption of available resources and energy, the MAE should be optimized according to maximum caffeine yield using a Box–Behnken design. According to this matrix, 17 extractions were carried out to optimize this process with three extraction parameters (factors). The design matrix was generated in software and the order of extractions was randomized to minimize the effects of unexpected variability on the system response (Table 2). The factor levels were chosen based on our knowledge of the microwave-assisted extraction process. A longer exposure of plant material to the effect of microwave irradiation can lead to the degradation of the bioactive compounds present in it.

The central point of the BBD representing the combination of the mean levels of the factors was repeated five times to statistically analyze the proposed regression model. The caffeine yield ranged between 0.17 and 1.01 g/100 g of the dry weight (d.w.). The lowest yield of caffeine (0.17 g/100 g d.w.) was noticed for experimental run 6, representing the combination of the following conditions: extraction time, 2 min; liquid-to-solid ratio, 5 mL/g; and microwave power, 465.5 W. The highest yield of caffeine (1.01 g/100 g d.w.) was obtained for experimental run 17, which had a combination of the following extraction conditions: extraction time, 2 min; liquid-to-solid ratio, 15 mL/g; and a microwave power of 465.5 W. The obtained caffeine yield was fitted with different polynomial equations to obtain the best possible agreement between the experimental and predicted data. Based on the statistical analysis of the used regression models depicted in Table 3, the second-order polynomial equation was selected as the most suitable for the prediction of caffeine yield.

The analysis of variance (ANOVA test) for the proposed regression model performed at a confidence level of 95% is presented in Table 4. As part of this analysis, the *p*- and *F*-values of all of the terms of the equation were determined. Statistically significant terms were considered to be all of those whose *p*-value was less than 0.05. The statistically significant terms of the polynomial equation were *B*, *AB*, *A*^2^, and *C*^2^. Other statistically insignificant terms can be excluded from the polynomial equation to improve the predictive abilities of the regression model, but after analysis, it was concluded that they should not be excluded. The *F*-value of the model was statistically significant, which was desirable when setting up a regression model. The *F*-value of the lack-of-fit of 0.02 is a statistically insignificant value in relation to the pure error. This value was the second confirmation of model adequacy. The value of the coefficient of variation was 14.7%. Adequate precision as a measure of the S/N ratio was 14.14. Having in mind this value was higher than the threshold value of 4 [22], the model signal can be considered adequate. Also, the coefficient of determination (R^2^) of 0.9636 indicated that 96.36% of the variation in caffeine yield can be predicted by the regression model. The predicted R^2^ of 0.9361 agreed with the adjusted R^2^ of 0.9168 since the difference between them was less than 0.2. The obtained data of ANOVA analysis indicated that the proposed regression model was valid and can be used for navigation in the designed space.

The regression model describing the extraction process can be presented in terms of coded values as follows (Equation (3)):

(3) $$Y=0.273-0.018A+0.179B-0.052C-0.230AB-0.054AC+0.005BC+0.271A^{2}+0.020B^{2}+0.220C^{2}$$

where *Y* is the caffeine yield, *A* is the extraction time, *B* is the liquid-to-solid ratio, and *S* is the microwave power.

The terms of the polynomial equation that had the highest value of the regression coefficient significantly influenced the caffeine yield. The positive regression coefficient influenced the increase in the caffeine yield and vice versa. According to these guidelines, the liquid-to-solid ratio had the greatest effect, followed by microwave power and extraction time. Of the linear terms, the only increase in the liquid–solid ratio led to a significant increase in caffeine yield, while in other cases this value decreased. The results obtained in this study are in accordance with the available literature data. Tran et al. [23] also confirmed that in terms of individual effects, the liquid-to-solid ratio significantly influences the yield of caffeine extracted from coffee pulp using ethanol. In the closed-vessel MAE system, Pettinato et al. [24] also showed that the yield of antioxidants, including caffeine, from spent coffee grounds decreases upon increasing the extraction time.

**Figure 2:**
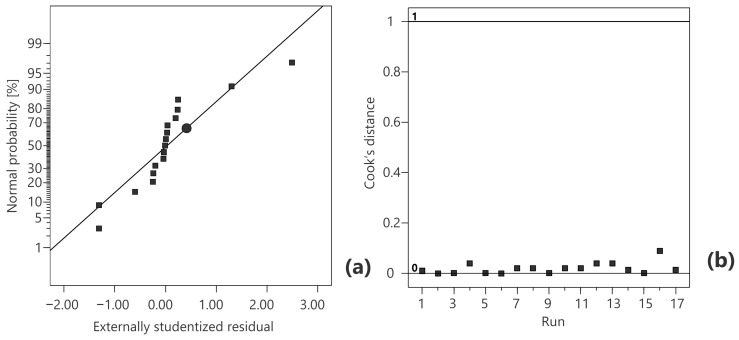
Normal probability plot (**a**) and Cook’s distance (**b**) for the second-order polynomial model.

The normal probability plot for the regression model is depicted in Figure 2a. If the externally studentized residuals follow a normal distribution, the data on the plot have almost a linear dependency [25]. Given that the graph was S-shaped, it can be concluded that the residuals had a smaller variance than expected. In Figure 2b, Cook’s distance graph indicated that there were no outliers during their modeling and all data were less than the threshold value of 1 [26].

**Figure 3:**
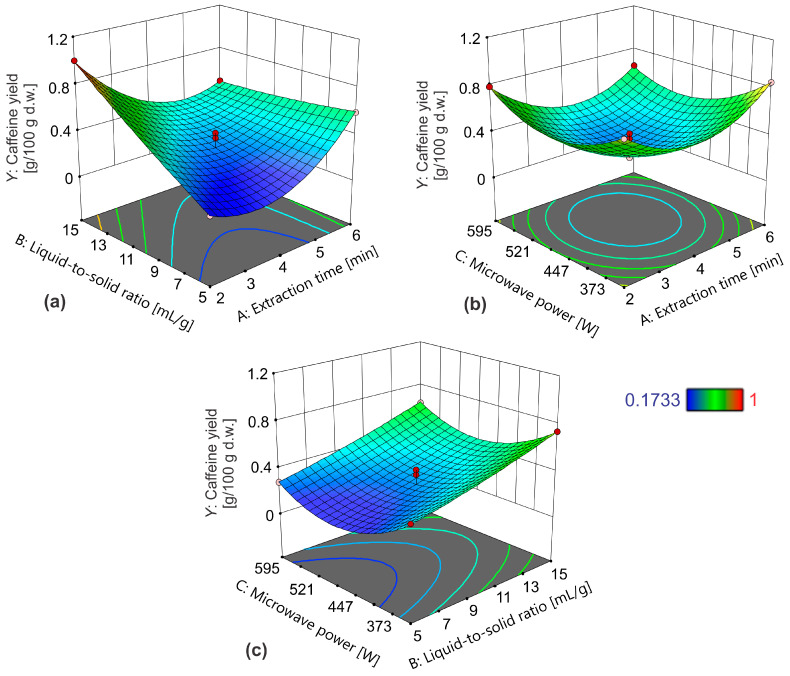
The effect of (**a**) extraction time and liquid-to-solid ratio at a microwave power of 465.5 W; (**b**) extraction time and microwave power at a liquid-to-solid ratio of 10 mL/g; and (**c**) the liquid-to-solid ratio and microwave power on caffeine yield for the extraction time of 4 min.

The three-dimensional plots were generated to visualize the effect of extraction parameters on caffeine yield (Figure 3). The effect of extraction time and the liquid-to-solid ratio on caffeine yield at 465.5 W of microwave power is presented in Figure 3a. A strong interaction was noticed between the observed extraction parameters. At the lower liquid-to-solid ratios, the caffeine yield increased upon increasing the extraction time. The caffeine yield decreased at higher liquid-to-solid ratios. The effect of the liquid-to-solid ratio was negligibly small at longer extraction times. For shorter extraction times, the caffeine yield was significantly increased upon increasing the liquid-to-solid ratios. The results are consistent with the literature data where it was also observed that a longer extraction time and lower liquid-to-solid ratio increase the yield of extracted caffeine from roasted coffee using the closed-vessel MAE system [13]. The significant interactive effects between the extraction time and liquid-to-solid ratio on the yield of extracted caffeine from coffee pulp were also noticed by Tran et al. [23]. In Figure 3b, a strong interaction can also be observed between the extraction time and the microwave power. In this dependence, the minimum caffeine yield was noticed at medium factor levels of the observed extraction parameters. Regarding the interactive impact of these effects, Tran et al. [23] concluded that they had no significant impact on the yield of extracted caffeine from coffee pulp.

The effect of the liquid-to-solid ratio and microwave power on the caffeine yield for the extraction time of 4 min is depicted in Figure 3c. This dependence indicates a decrease in caffeine yield at the medium factor level of microwave power. The increase in caffeine yield also occurred upon increasing the liquid-to-solid ratio, regardless of microwave power. This was expected since a high liquid-to-solid ratio increases the concentration gradient, resulting in a better diffusion process [27]. Tran et al. [23] also showed that this interaction influence had no significant effect on the yield of extracted caffeine from coffee pulp.

### 3.2. Optimization and Validation of Microwave-Assisted Extraction of Caffeine from Roasted Coffee Beans

The optimization procedure of a specific system can be formally divided into two levels. At the first level, defining the problem and establishing the exact relationships of factors and solutions in the conditions under which the system should function is necessary. The second level represents the choice of one of the known methodologies or the development of a new methodology for solving the problem, where the defined problem must satisfy the formal and mathematical limitations of the chosen method. Also, the chosen method must enable reliable and accurate determination of optimal solutions in the simplest and fastest way possible. A numerical optimization method was used to optimize the open-vessel MAE system of caffeine from coffee beans. Before applying this method, the degree of importance of all factors was medium (+++). After the optimization studies, the following extraction conditions were proposed: extraction time, 2.0 min; liquid-to-solid ratio, 15 mL/g; and microwave power, 500 W. The extraction was carried out under these conditions to verify their validity. The obtained experimental value of caffeine yield (0.98 g/100 g d.w.) was in close agreement with the predicted value (1.01 g/100 g d.w.) by the regression model. The low difference between these values indicated that the regression model was valid and could be used to predict the caffeine yield. Under these optimal conditions, the TEC was 1.7 kWh/100 g of purified caffeine.

The recommended optimal extraction time of 2 min was in line with the literature data reported by Lopes et al. [13]. Andrade et al. [28] obtained the highest extraction yield of caffeine (6.45 mg/g) from spent coffee grounds using a supercritical fluid extraction method. Tran et al. [23] optimized the closed-vessel MAE system of caffeine from coffee pulp and obtained the following conditions: extraction time, 85 min; liquid-to-solid ratio, 1 mL/g, with 42.5% (*v*/*v*) ethanol; and microwave power, 1000 W. Under the given conditions, the caffeine yield was about 5.5 mg/g d.w. According to Equation (2), the TEC in their study was found to be 25.9 MWh/100 g of extracted caffeine, which was about 15,000 times higher than the TEC in our study. Pettinato et al. [24] optimized the closed-vessel MAE system of antioxidants from spent coffee grounds. They achieved the highest caffeine yield (32 mg/L) at an extraction temperature of 150 °C; extraction time, 90 min; 54% (*v*/*v*) ethanol; and microwave power, 500 W. The TEC in their study was about 2.1 MW/100 g of extracted caffeine, which was about 1200 times higher than in our study. Based on the obtained results in our study, it can be concluded that the proposed procedure has an advantage compared with the procedures available in the literature in terms of a shorter extraction time of about 45 times and a lower TEC of about 1200–15,000 times using the microwave-assisted extraction procedures and using simpler equipment.

### 3.3. Structural Characterization and Purity of Isolated Caffeine

**Figure 4:**
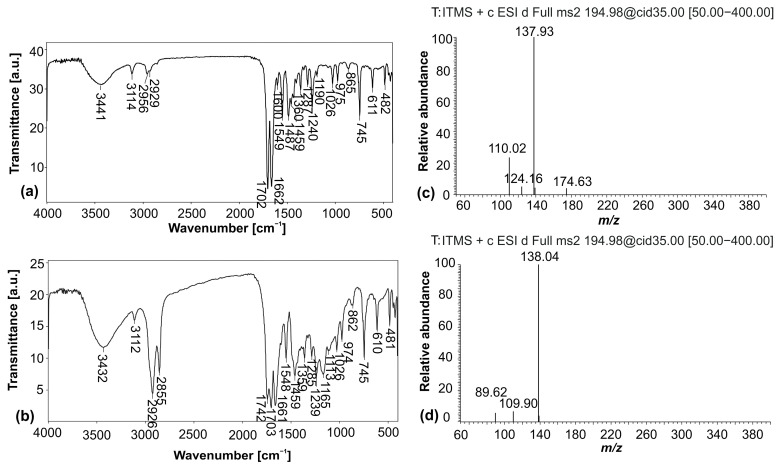
FTIR spectra of standard caffeine (**a**) and isolated caffeine (**b**); mass spectra of standard caffeine (**c**) and isolated caffeine (**d**) recorded in the positive mode at a retention time of 0.97 min.

The FTIR spectra of the standard and isolated caffeine from coffee beans are shown in Figure 4. In the spectrum of standard caffeine (Figure 4a), a broad band at 3441 cm^−1^ originated from the valence vibration of the N–H bond, while the bands at 3114 cm^−1^ and 2956 cm^−1^ corresponded to the valence vibrations of aromatic C–H bonds. The bands at 1702 cm^−1^ and 1662 cm^−1^ were the result of valence vibrations of the –C=N and C=O bonds of the aromatic ring, respectively. Rajam et al. [29] also obtained the characteristic bands of caffeine as presented in this study. Completely identical bands with some shifting in wavenumbers and intensity can be observed in the spectrum of isolated caffeine (Figure 4b). The bands of N–H, aromatic C–H, –C=N, and C=O valence vibrations shifted slightly to 3432 cm^−1^, 3112 cm^−1^ and 2926 cm^−1^, and 1703 cm^−1^ and 1661 cm^−1^, respectively. Based on these results, it can be concluded that the obtained sample of caffeine was successfully isolated and purified.

Determining the purity of isolated bioactive compounds is of crucial importance for their further use. In this study, the UHPLC-ESI-MS/MS method was applied to analyze the isolated caffeine sample. The calibration curve of standard caffeine was necessary to construct in order to determine the caffeine content in the analyzed sample. The method linearity was achieved in a caffeine concentration range of 5–40 µg/mL. The intercept and slope of the constructed calibration curve for caffeine, including the LOD and LOQ, are given in Table 5. The high calculated value of caffeine purity of 96.3% indicated that the proposed procedure for its isolation is suitable for obtaining a high-quality substance. Tello et al. [30] obtained a purity in the range of 61–77% for caffeine isolated from coffee husks using supercritical CO_2_ extraction. According to Pradeep and Rameshaiah [16], the purity was about 90% for caffeine isolated from coffee beans using dichloromethane solvent and including distillation and recrystallization purification processes. The mass spectra of standard and isolated caffeine at the retention time of 0.97 min are presented in Figure 4c,d.

## 4. Conclusions

The second-order polynomial model was successfully applied to model and describe the MAE of caffeine from roasted coffee beans, given that the highest coefficient of determination of 0.9636 was obtained for this model compared to other analyzed models. The results of the ANOVA test showed that of the linear terms, the liquid-to-solid ratio had the greatest influence on the caffeine yield, followed by the microwave power and the extraction time. Of all of the linear terms, only the liquid-to-solid ratio has a positive effect on the caffeine yield. Using the numerical optimization method, the following optimal conditions were obtained: extraction time, 2.0 min; liquid-to-solid ratio, 15 mL/g, and microwave power, 500 W. Based on the results of FTIR and UHPLC-ESI-MS/MS analysis, it can be concluded that caffeine was more successfully isolated and purified than other compounds previously extracted from coffee beans. The determined purity of isolated caffeine was higher than those obtained according to the other available procedures indicating the high quality of the substance. This study’s contribution was applying the green extraction technique, reducing extraction time and consumed energy. Further research will focus on the purification of the resulting caffeine for use as an active ingredient in pharmaceutical products.

## Figures and Tables

**Table 1:** Factor levels of analyzed extraction parameters.

Factors	Coded Value		
	−1	0	+1
	Actual Value		
Extraction time [min]	2	4	6
Liquid-to-solid ratio [mL/g]	5	10	15
Microwave power [W]	336	465.5	595

**Table 2:** The Box–Behnken design matrix with randomly ordered data. The actual and coded factor levels are shown in parentheses. The central point of the used design and standard order (Std.) of carried-out extractions are depicted. The caffeine yield is expressed as grams per 100 g of dry weight.

Std.	Run	*A*: Extraction Time [min]	*B*: Liquid-to-Solid Ratio [mL/g]	*C*: Microwave Power [W]	*Y*: Caffeine Yield [g/100 g d.w.]	
					Experimental ^a^	Predicted
16	1 *	4 (0)	10 (0)	465.5 (0)	0.23 ± 0.01	0.27
4	2	6 (+1)	15 (+1)	465.5 (0)	0.50 ± 0.02	0.49
7	3	2 (−1)	10 (0)	595.0 (+1)	0.79 ± 0.03	0.78
14	4 *	4 (0)	10 (0)	465.5 (0)	0.19 ± 0.01	0.27
6	5	6 (+1)	10 (0)	336.0 (−1)	0.85 ± 0.02	0.85
1	6	2 (−1)	5 (−1)	465.5 (0)	0.17 ± 0.01	0.17
12	7	4 (0)	15 (+1)	595.0 (+1)	0.64 ± 0.03	0.65
5	8	2 (−1)	10 (0)	336.0 (−1)	0.77 ± 0.03	0.78
10	9	4 (0)	15 (+1)	336.0 (−1)	0.74 ± 0.02	0.74
8	10	6 (+1)	10 (0)	595.0 (+1)	0.65 ± 0.04	0.64
9	11	4 (0)	5 (−1)	336.0 (−1)	0.40 ± 0.02	0.39
13	12 *	4 (0)	10 (0)	465.5 (0)	0.19 ± 0.01	0.27
15	13 *	4 (0)	10 (0)	465.5 (0)	0.36 ± 0.01	0.27
2	14	6 (+1)	5 (−1)	465.5 (0)	0.59 ± 0.02	0.60
11	15	4 (0)	5 (−1)	595 (+1)	0.28 ± 0.01	0.28
17	16 *	4 (0)	10 (0)	465.5 (0)	0.40 ± 0.02	0.27
3	17	2 (−1)	15 (+1)	465.5 (0)	1.00 ± 0.07	0.99

* Central point of Box–Behnken design; ^a^ Mean ± standard deviation of three measurements.

**Table 3:** Statistical data for different polynomial regression models for modeling caffeine yield.

Model	Lack of Fit	Adjusted R^2^	Predicted R^2^
Linear model	0.02565	0.083	−0.269
A model with two-factor interactions	0.024206	0.136	−0.570
Second-order model	0.996154	0.917	0.936
Cubic model		0.856	

**Table 4:** Analyses of variance for the second-order polynomial model.

	Sum of Squares	Degree of Freedom	Mean Value of Sum of Squares	*F*-Value	*p*-Value
Model	1.0552	9	0.1172	20.5956	0.00031 *
*A*—Extraction time	0.0026	1	0.0026	0.4617	0.51867
*B*—Liquid-to-solid ratio	0.2553	1	0.2553	44.8502	0.00028 *
*C*—Microwave power	0.0213	1	0.0213	3.7363	0.09451
*AB*	0.2124	1	0.2124	37.3058	0.00049 *
*AC*	0.0117	1	0.0117	2.0616	0.19419
*BC*	9.18 × 10^−5^	1	9.18 × 10^−5^	0.0161	0.90250
*A* ^2^	0.3103	1	0.3103	54.5127	0.00015 *
*B* ^2^	0.0018	1	0.0018	0.3089	0.59564
*C* ^2^	0.2038	1	0.2038	35.8057	0.00055 *
Residual	0.0398	7	0.0057		
Lack-of-fit	0.0005	3	0.0002	0.0181	0.99615
Pure error	0.0393	4	0.0098		
Corrected sum of squares	1.0950	16			
Standard deviation	0.0754		R^2^	0.9636	
Mean value	0.5136		Adjusted R^2^	0.9168	
Coefficient of variation (%)	14.7		Predicted R^2^	0.9361	
			Adequate precision	14.14	

*—Statistically significant value (*p* < 0.05).

**Table 5:** Linear regression parameters for the calibration curves of caffeine. The data are presented as mean ± standard deviation (SD). The LOD and LOQ were determined based on the standard deviation of the intercept and the slope of the calibration curve.

Parameters	Values
Slope	2485.90 ± 138.03
Intercept	−1952.17 ± 3181.42
R^2^	0.994
Adjusted R^2^	0.991
LOD (μg/mL)	4.22
LOQ (μg/mL)	12.66

## Data Availability

The original contributions presented in the study are included in the article, further inquiries can be directed to the corresponding author.
